# A Stitch in Time - Emergency Repair of post-ROSC patient with Acute Traumatic Cardiac Tamponade following Penetrating Chest Trauma

**DOI:** 10.12669/pjms.41.9.11163

**Published:** 2025-09

**Authors:** Muhammad Saad Sikander, Osama Abdul Mateen, Muhammad Saad Farooq, Tariq Mehmood

**Affiliations:** 1 Muhammad Saad Sikander, FCPS Trainee in Anesthesiology Department of Anesthesiology, Combined Military Hospital, Rawalpindi, Pakistan; 2 Osama Abdul Mateen, FCPS Trainee in Anesthesiology Department of Anesthesiology, Combined Military Hospital, Rawalpindi, Pakistan; 3 Muhammad Saad Farooq, FCPS Trainee in Anesthesiology Department of Anesthesiology, Combined Military Hospital, Rawalpindi, Pakistan; 4 Tariq Mehmood, FCPS Anesthesiology, FCPS Pain Medicine Department of Anesthesiology, Combined Military Hospital, Rawalpindi, Pakistan

**Keywords:** CPR, Pericardiocentesis, Shock, Tamponade

## Abstract

A 22-year-old male was brought to the ER with multiple chest stab wounds. The patient arrived in Grade-III shock. He collapsed shortly after arriving and needed CPR and was recovered. Blood transfusions were beginning immediately and bilateral chest tubes were passed. Despite these efforts, he had another cardiac arrest and was revived with another CPR. An eFAST scan, ECG and portable X-Ray confirmed severe Cardiac tamponade with bilateral Hemothorax. A 20 cc pericardiocentesis was performed and dobutamine infusion was started. He was rushed to OT where emergency evacuation of tamponade along with repair of injuries was performed. He was subsequently shifted to ICU where he received post-operative care and was discharged on tenth post-operative day.

## INTRODUCTION

Cardiac tamponade is a disease defined by the impairment of cardiac filling resulting from pericardial effusion. It presents as a spectrum of hemodynamic abnormalities, resulting in a clinical presentation that is variable rather than absolutely dichotomous. Severe instances of cardiac tamponade are characterized by an effusion diameter greater than 2 cm or the observation of a right ventricular diastolic collapse via echocardiography.^1^ Clinically, Beck’s triad may be observed, comprising muffled heart sounds, elevated jugular venous pressure, and hypotension; however, only around one-third of patients display this combination.^2^ These individuals often have pulsus paradoxus which is an exaggerated fall of blood pressure of more than 10 mmHg on inspiration. In instances of trauma, hematoma formation transpires rapidly, predominantly resulting from direct cardiac injury inflicted by penetrating thoracic wounds, particularly to the left of the sternum. These injuries have a significant mortality rate, with numerous patients failing to survive till hospital.^3^ This report delineates the case of a patient who sustained penetrating chest trauma resulting in cardiac tamponade and significant hemothorax and describes the effective care of these injuries.

## CASE PRESENTATION

A 22-year-old male with no known comorbidities was taken to the emergency department with several stab wounds to the chest incurred during a street altercation. Upon arrival, the patient had an altered state of consciousness, cold extremities, a thready pulse of 133, and a Mean Arterial Pressure of 55. The physical examination revealed bilateral coarse crackles in the chest and diminished heart sounds. Shortly after arrival, the patient collapsed, requiring Cardio-Pulmonary Resuscitation, which successfully reinstated sinus rhythm after 10 minutes. The patient underwent intubation and was placed on mechanical ventilation during this period. Large-bore intravenous catheters were swiftly inserted, and blood transfusion was initiated without delay.

**Fig.1:**
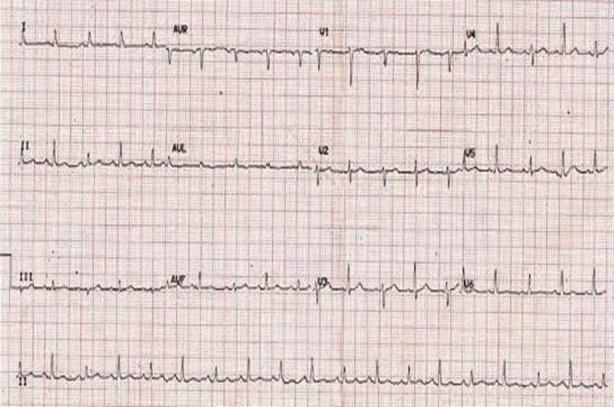
ECG showing sinus tachycardia with electrical alternans.

**Fig.2:**
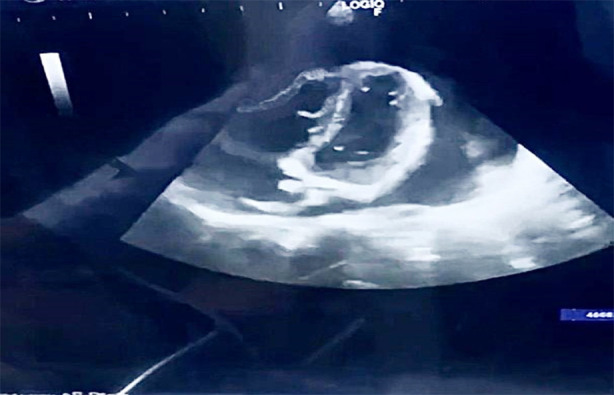
Subxiphoid view showing cardiac tamponade with bowing of RV free wall.

**Fig.3:**
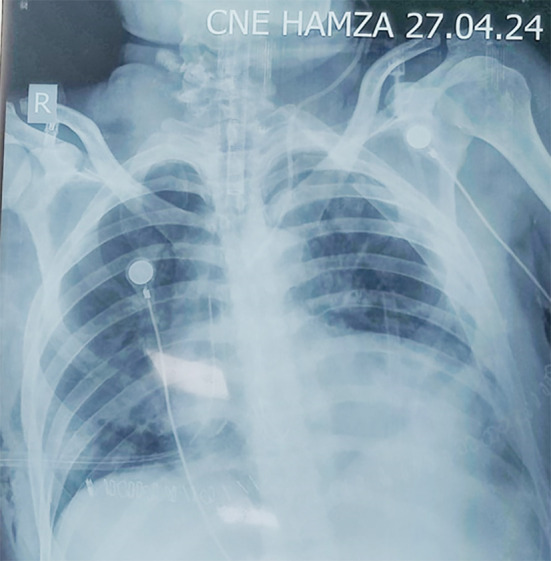
Bilateral chest tubes in place with enlarged cardiac silhoutte.

Chest tubes were placed to evacuate bilateral hemothorax. Notwithstanding these treatments, the patient suffered another episode of cardiac arrest necessitating CPR, resulting in successful resuscitation once more. The Extended Focused Assessment with Sonography for Trauma (eFAST) scan revealed cardiac tamponade along with by right ventricular diastolic collapse in the subxiphoid view and stunned myocardium with a low ejection fraction of 30%. The electrocardiogram performed upon arrival revealed sinus tachycardia accompanied by low-voltage QRS complexes and electrical alternans. The portable chest X-ray verified bilateral hemothorax and an enlarged cardiac silhouette (Fig.1, 2, and 3, respectively). A pericardiocentesis of 20 cc was conducted to alleviate tamponade while the patient was being prepared for surgery. Inotropic support was initiated with dobutamine.

A prompt coordination was formed with the Anesthesiology and Cardiothoracic Surgery departments, and the patient was shifted to the Operating Theatre, where the Single Lumen Endotracheal tube was replaced with a Double Lumen Endobronchial tube for one-lung ventilation to improve surgical access. An arterial line was placed for invasive blood pressure monitoring, and the Massive Transfusion Protocol was activated due to the patient’s hemodynamic status and the anticipated surgical interventions. The patient received a clamshell thoracotomy, pericardiotomy, and cardiac repair. An enlarged, sac-like pericardium was observed, which, following incision, disclosed frank blood. Subsequently, the patient’s hemodynamic state improved. Right lung repair and diagnostic laparotomy was also performed thereafter. During the operation, the patient encountered many instances of atrial fibrillation and ventricular tachycardia accompanied by hypotension, which was managed with cardioversion. During the operation, the patient experienced a transient episode of cardiac arrest, which was addressed by cardiac massage.

The patient was transferred to the intensive care unit for critical care management, including mechanical ventilation, inotropic support, and pain management. Despite the gravity of the injuries, the patient was extubated on the first postoperative day and then weaned off inotropic support the next day. The pericardial drain was removed on the fifth post-operative day along with the patient being mobilized, and the patient was discharged from the hospital on the tenth post-operative day.

## DISCUSSION

In most patients, mild to moderate tamponade is treated conservatively, however pericardiocentesis gives a more definitive solution. Even removing a modest amount of blood can significantly enhance heart function. The optimal therapeutic method for severe cardiac tamponade, however, is surgical evacuation of blood from the pericardial space.^4^ Several temporary methods that can buy time for the patient are typically targeted at boosting the stroke volume. They include boosting intravascular volume using crystalloids or blood, using inotropes, and correcting acidosis.^5^

This case emphasizes the need of early detection and management of cardiac tamponade, as well as the multidisciplinary approach to a patient with life-threatening injuries. Prompt treatment in the emergency department was critical to the patient’s survival.

The expansion of intravenous volume with blood transfusions were essential to treat the patient’s hemodynamic instability and maintaining preload to enhance cardiac output. The placement of chest tubes drained the bilateral hemothorax, and shifting the patient to OT for definitive surgery was also an important step. The intraoperative management necessitated a high level of skill and coordination between the anesthesiology and surgical teams. General anesthesia and positive pressure ventilation can lead to cardiovascular collapse in the event of significant tamponade.^6^ The primary objectives are to maintain proper cardiac output and blood pressure. Myocardial depression caused by intravenous and inhalational anesthetics is prevented, particularly during induction. Invasive blood pressure monitoring and large-bore IV access are also required. After relieving tamponade, a period of severe hypertension ensues, which should be anticipated and addressed accordingly.^7^ This case also had an added problem of a stunned myocardium, which occurs in the post-Return of Spontaneous Circulation ROSC phase. One-lung ventilation was also difficult in this patient, reducing right ventricle output and deteriorating cardiac output, which was managed with occasional bilateral ventilation as clinically needed.

The frequency of cardiac arrests needed early detection and care which necessitated constant communication between surgeons and anesthesiologists and represented a collaborative effort to improve the patient’s condition. A clamshell thoracotomy gave sufficient exposure to repair both cardiac and pulmonary damage in one incision. Diagnostic laparotomy was performed to check for any injuries extending into the abdomen. Repairing cardiac lacerations was extremely difficult because dysrhythmias significantly worsened the patient’s condition. The patient’s remarkable recovery underscores the effectiveness of early recognition, the timely interventions and professional expertise of the healthcare staff. The management in the emergency department, coupled with swift surgical action and comprehensive intraoperative and postoperative care, were crucial for the patient’s survival. It illustrates the critical role of a multidisciplinary strategy in addressing severe trauma cases, as well as the imperative for prompt evaluation, diagnosis, and treatment.

### Authors’ Contribution:

**MSS:** Conceived, designed, drafted the manuscript and is responsible for integrity of research.

**OAM & MSF:** Verified data accuracy, literature review, oversaw case management.

**TM:** Analyzed and organized patient data, oversaw and approved final manuscript.
